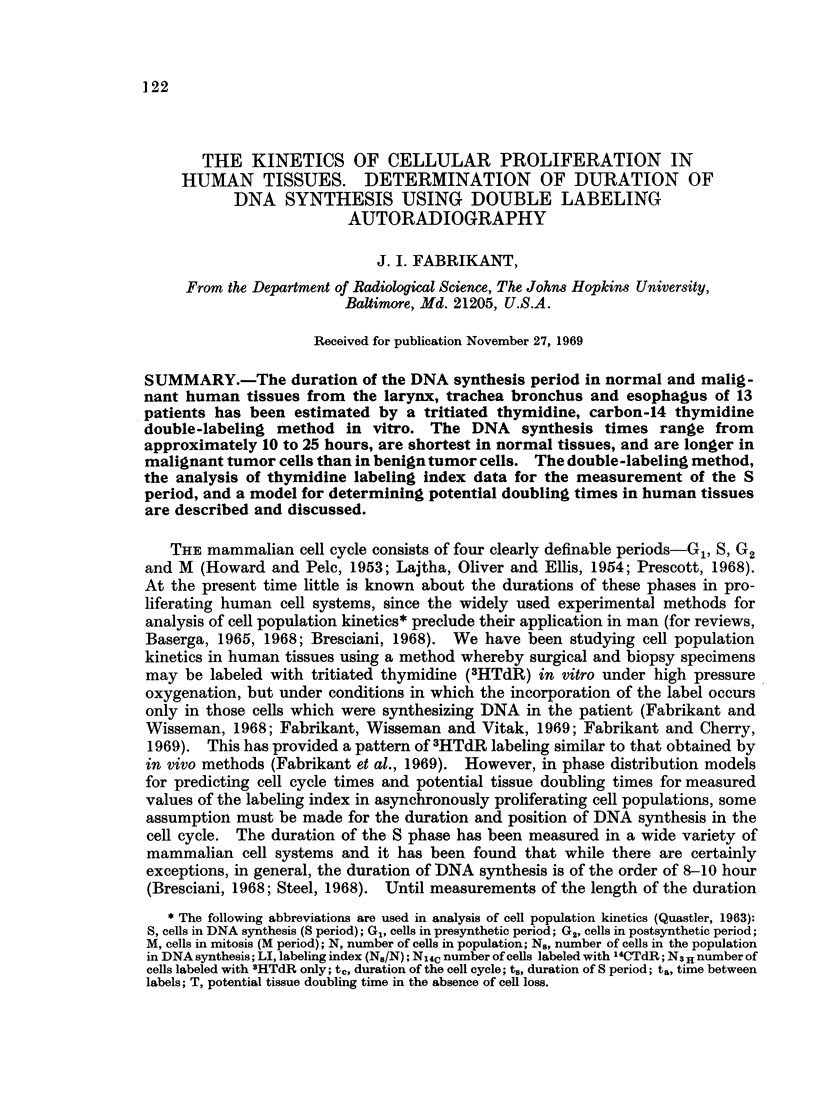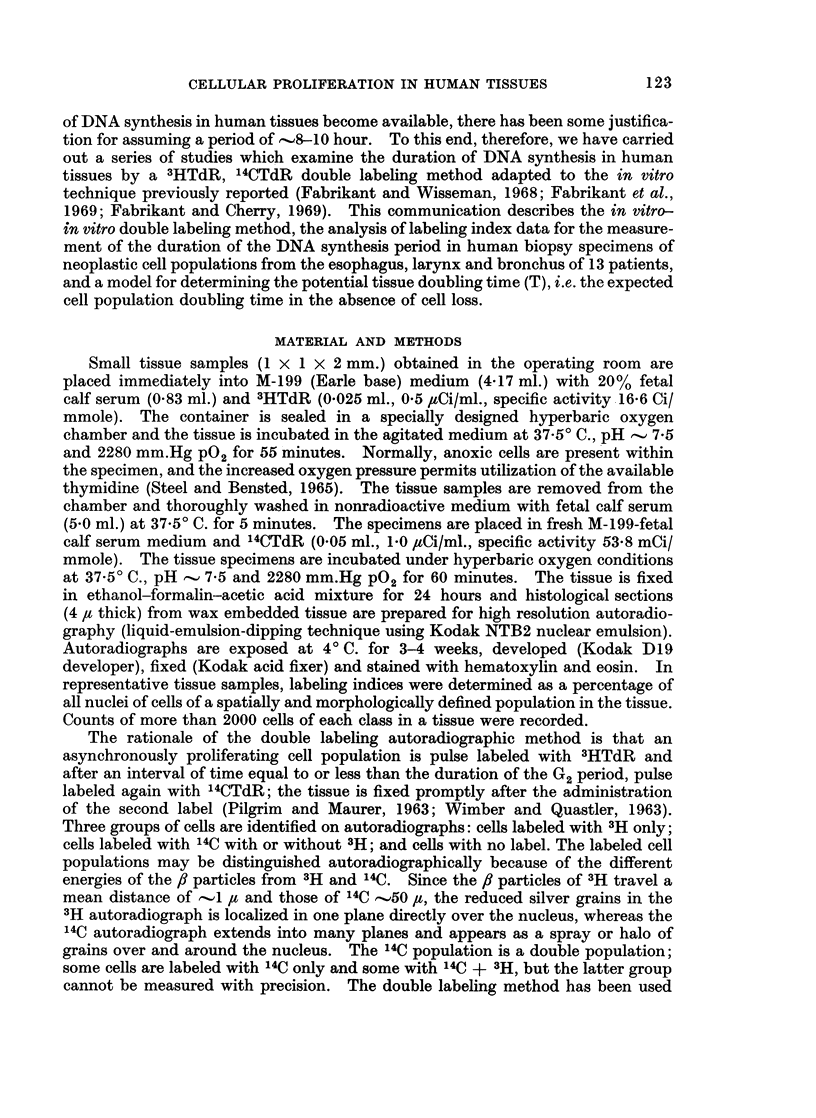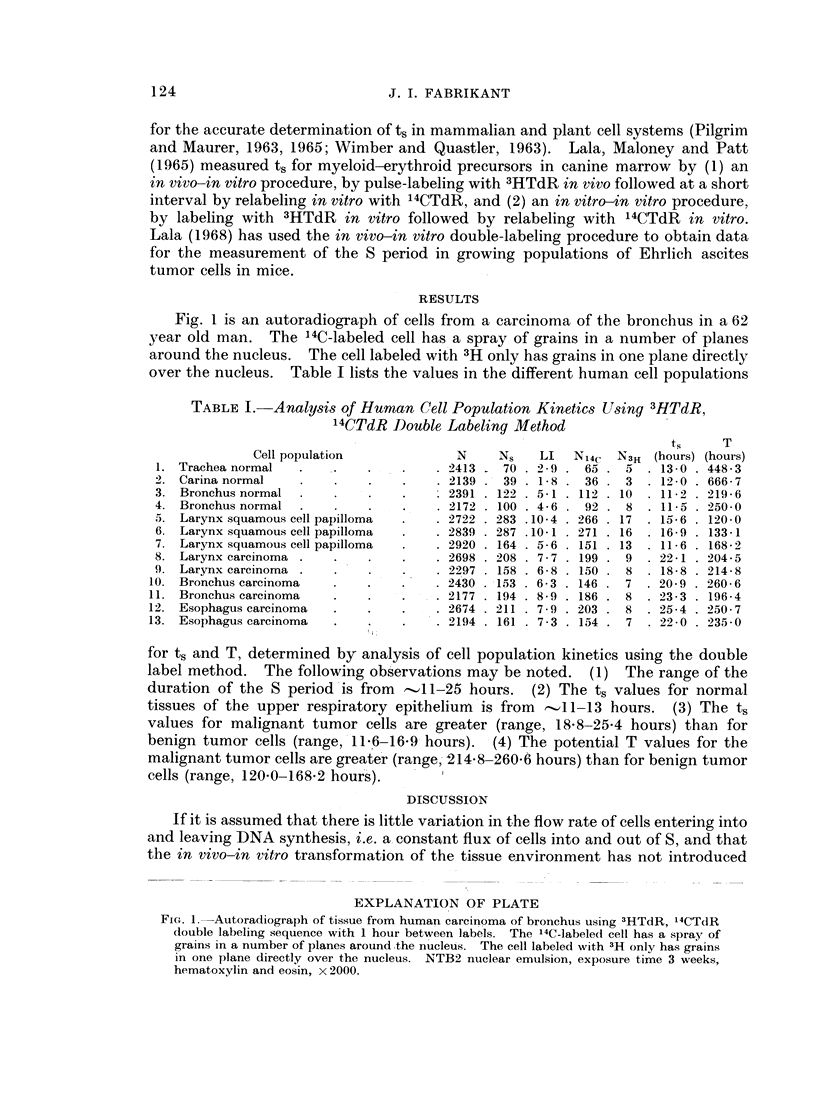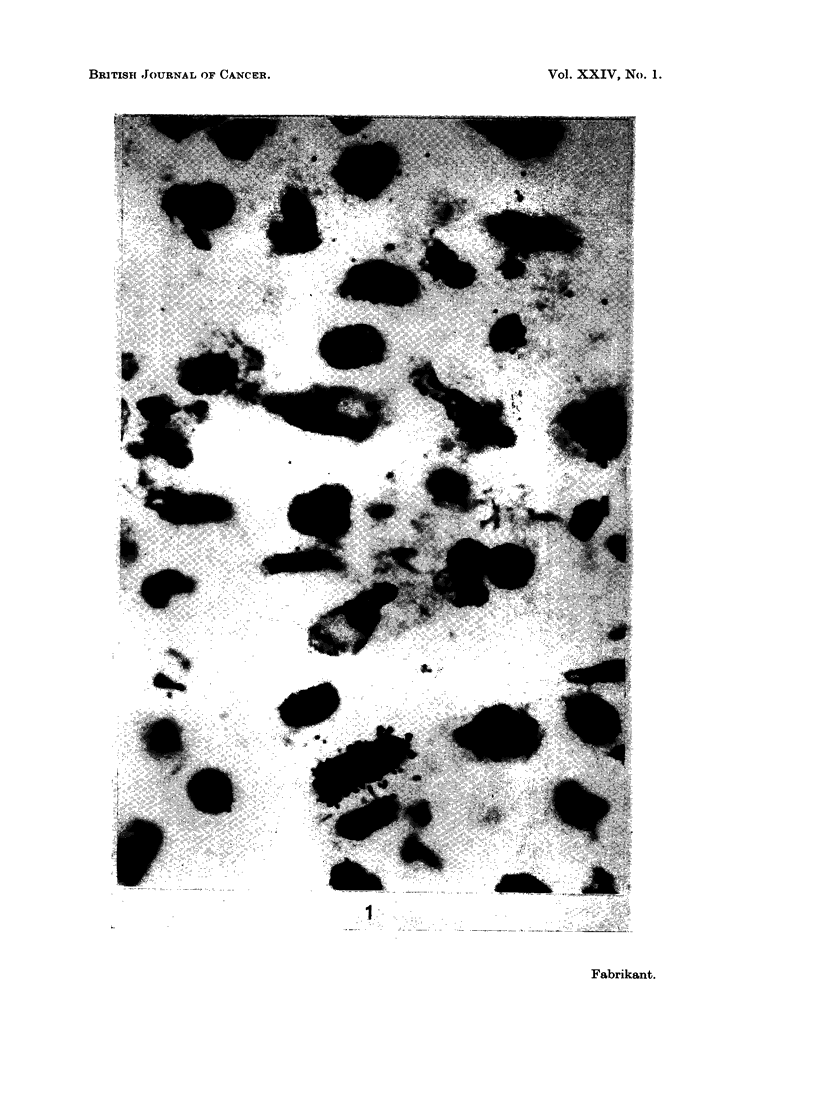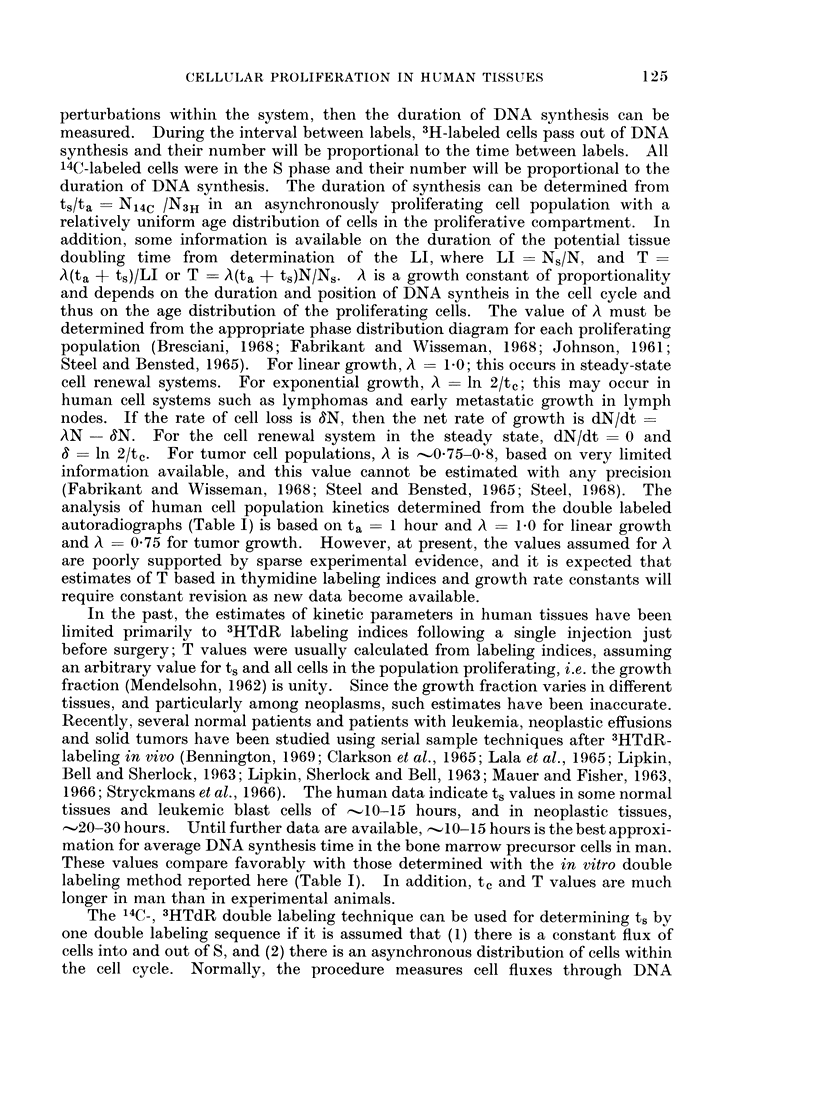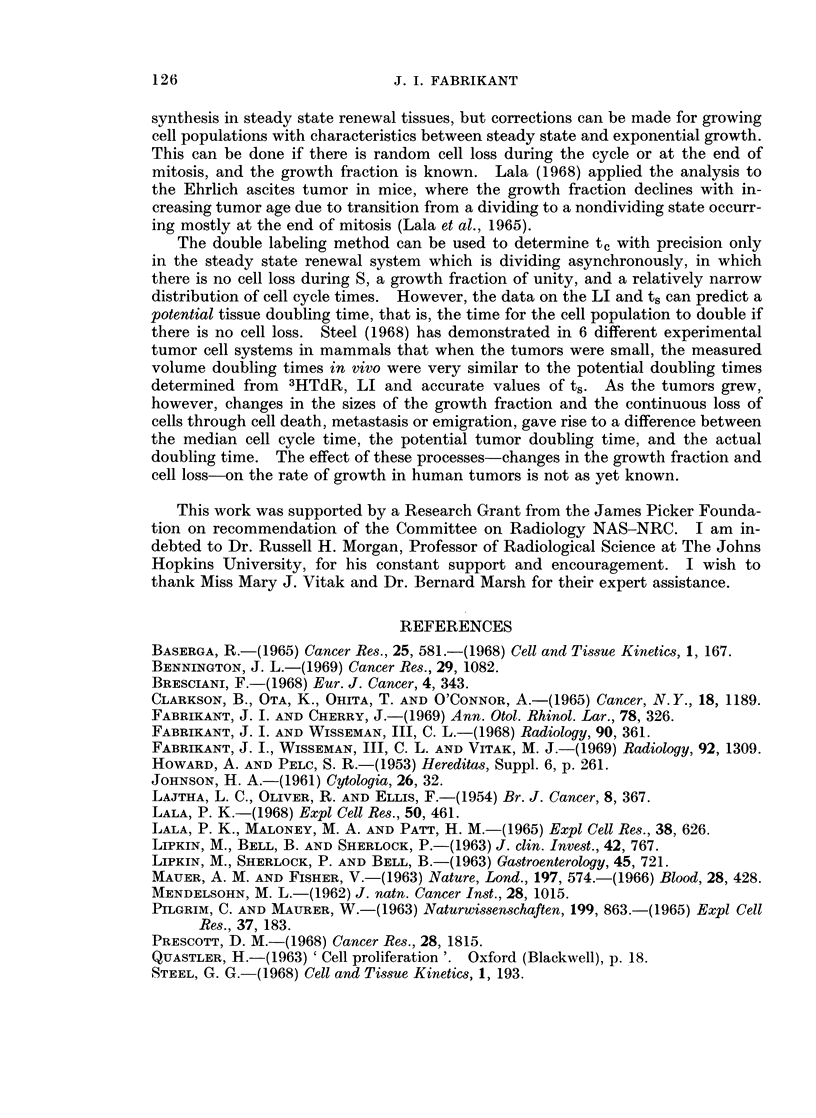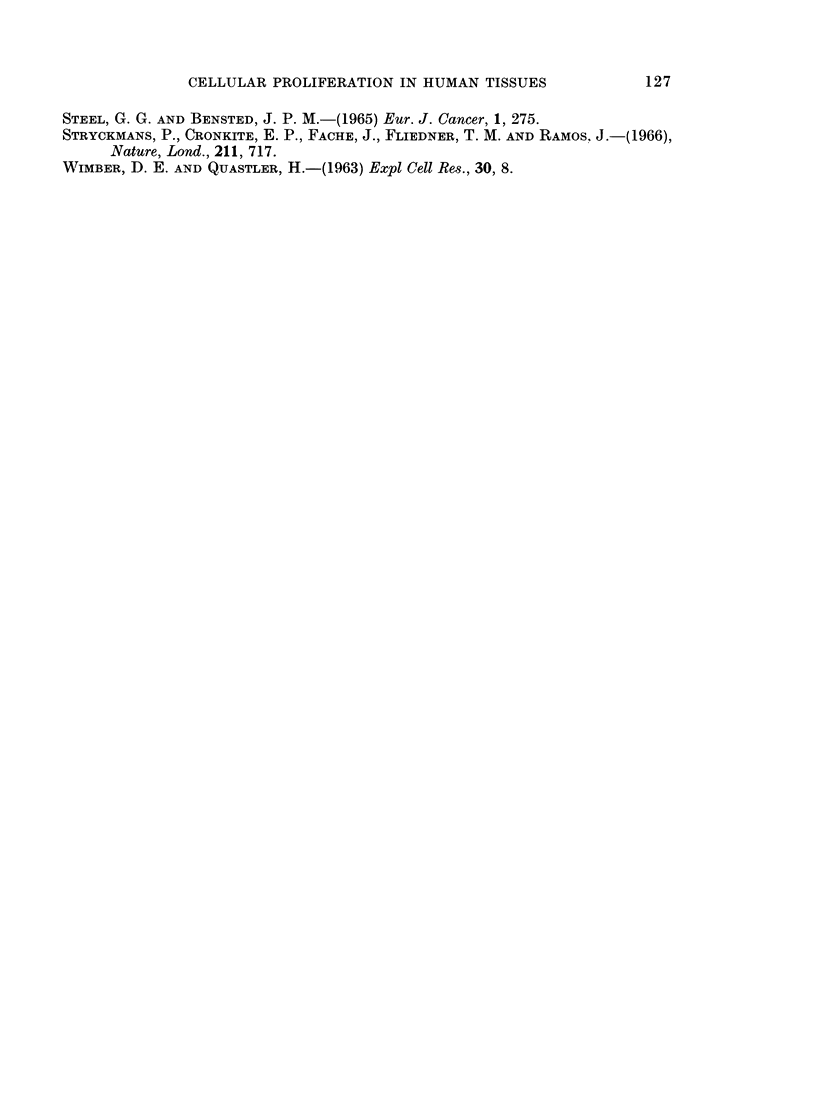# The Kinetic of Cellular Proliferation in Human Tissues. Determination of Duration of DNA Synthesis Using Double Labeling Autoradiography

**DOI:** 10.1038/bjc.1970.15

**Published:** 1970-03

**Authors:** J. I. Fabrikant

## Abstract

**Images:**


### THE KINETICS OF CELLULAR PROLIFERATION IN HUMAN TISSUES. DETERMINATION OF DURATION OF DNA SYNTHESIS USING DOUBLE LABELING AUTORADIOGRAPHY

J. I. FABRIKANT,

*From the Department of Radiological Science, The Johns Hopkins University, Baltimore, Md.* 21205, *U.S.A.*

Received for publication November 27, 1969

**SUMMARY.—The duration of the DNA synthesis period in normal and malignant human tissues from the larynx, trachea bronchus and esophagus of 13 patients has been estimated by a tritiated thymidine, carbon-14 thymidine double-labeling method in vitro. The DNA synthesis times range from approximately 10 to 25 hours, are shortest in normal tissues, and are longer in malignant tumor cells than in benign tumor cells. The double-labeling method, the analysis of thymidine labeling index data for the measurement of the S period, and a model for determining potential doubling times in human tissues are described and discussed.**

THE mammalian cell cycle consists of four clearly definable periods—$G_1$, S, $G_2$ and M (Howard and Pelc, 1953; Lajtha, Oliver and Ellis, 1954; Prescott, 1968). At the present time little is known about the durations of these phases in proliferating human cell systems, since the widely used experimental methods for analysis of cell population kinetics* preclude their application in man (for reviews, Baserga, 1965, 1968; Bresciani, 1968). We have been studying cell population kinetics in human tissues using a method whereby surgical and biopsy specimens may be labeled with tritiated thymidine ($^3$HTdR) *in vitro* under high pressure oxygenation, but under conditions in which the incorporation of the label occurs only in those cells which were synthesizing DNA in the patient (Fabrikant and Wisseman, 1968; Fabrikant, Wisseman and Vitak, 1969; Fabrikant and Cherry, 1969). This has provided a pattern of $^3$HTdR labeling similar to that obtained by *in vivo* methods (Fabrikant *et al.*, 1969). However, in phase distribution models for predicting cell cycle times and potential tissue doubling times for measured values of the labeling index in asynchronously proliferating cell populations, some assumption must be made for the duration and position of DNA synthesis in the cell cycle. The duration of the S phase has been measured in a wide variety of mammalian cell systems and it has been found that while there are certainly exceptions, in general, the duration of DNA synthesis is of the order of 8–10 hour (Bresciani, 1968; Steel, 1968). Until measurements of the length of the duration

* The following abbreviations are used in analysis of cell population kinetics (Quastler, 1963): S, cells in DNA synthesis (S period); $G_1$, cells in presynthetic period; $G_2$, cells in postsynthetic period; M, cells in mitosis (M period); N, number of cells in population; $N_s$, number of cells in the population in DNA synthesis; LI, labeling index ($N_s$/N); $N_{14C}$ number of cells labeled with $^{14}$CTdR; $N_{3H}$ number of cells labeled with $^3$HTdR only; $t_c$, duration of the cell cycle; $t_s$, duration of S period; $t_a$, time between labels; T, potential tissue doubling time in the absence of cell loss.

of DNA synthesis in human tissues become available, there has been some justification for assuming a period of ~8–10 hour. To this end, therefore, we have carried out a series of studies which examine the duration of DNA synthesis in human tissues by a $^3$HTdR, $^{14}$CTdR double labeling method adapted to the *in vitro* technique previously reported (Fabrikant and Wisseman, 1968; Fabrikant *et al.*, 1969; Fabrikant and Cherry, 1969). This communication describes the *in vitro–in vitro* double labeling method, the analysis of labeling index data for the measurement of the duration of the DNA synthesis period in human biopsy specimens of neoplastic cell populations from the esophagus, larynx and bronchus of 13 patients, and a model for determining the potential tissue doubling time (T), *i.e.* the expected cell population doubling time in the absence of cell loss.

### MATERIAL AND METHODS

Small tissue samples (1 × 1 × 2 mm.) obtained in the operating room are placed immediately into M-199 (Earle base) medium (4·17 ml.) with 20% fetal calf serum (0·83 ml.) and $^3$HTdR (0·025 ml., 0·5 $\mu$Ci/ml., specific activity 16·6 Ci/mmole). The container is sealed in a specially designed hyperbaric oxygen chamber and the tissue is incubated in the agitated medium at 37·5° C., pH ~ 7·5 and 2280 mm.Hg $pO_2$ for 55 minutes. Normally, anoxic cells are present within the specimen, and the increased oxygen pressure permits utilization of the available thymidine (Steel and Bensted, 1965). The tissue samples are removed from the chamber and thoroughly washed in nonradioactive medium with fetal calf serum (5·0 ml.) at 37·5° C. for 5 minutes. The specimens are placed in fresh M-199-fetal calf serum medium and $^{14}$CTdR (0·05 ml., 1·0 $\mu$Ci/ml., specific activity 53·8 mCi/mmole). The tissue specimens are incubated under hyperbaric oxygen conditions at 37·5° C., pH ~ 7·5 and 2280 mm.Hg $pO_2$ for 60 minutes. The tissue is fixed in ethanol–formalin–acetic acid mixture for 24 hours and histological sections (4 $\mu$ thick) from wax embedded tissue are prepared for high resolution autoradiography (liquid-emulsion-dipping technique using Kodak NTB2 nuclear emulsion). Autoradiographs are exposed at 4° C. for 3–4 weeks, developed (Kodak D19 developer), fixed (Kodak acid fixer) and stained with hematoxylin and eosin. In representative tissue samples, labeling indices were determined as a percentage of all nuclei of cells of a spatially and morphologically defined population in the tissue. Counts of more than 2000 cells of each class in a tissue were recorded.

The rationale of the double labeling autoradiographic method is that an asynchronously proliferating cell population is pulse labeled with $^3$HTdR and after an interval of time equal to or less than the duration of the $G_2$ period, pulse labeled again with $^{14}$CTdR; the tissue is fixed promptly after the administration of the second label (Pilgrim and Maurer, 1963; Wimber and Quastler, 1963). Three groups of cells are identified on autoradiographs: cells labeled with $^3$H only; cells labeled with $^{14}$C with or without $^3$H; and cells with no label. The labeled cell populations may be distinguished autoradiographically because of the different energies of the $\beta$ particles from $^3$H and $^{14}$C. Since the $\beta$ particles of $^3$H travel a mean distance of ~1 $\mu$ and those of $^{14}$C ~50 $\mu$, the reduced silver grains in the $^3$H autoradiograph is localized in one plane directly over the nucleus, whereas the $^{14}$C autoradiograph extends into many planes and appears as a spray or halo of grains over and around the nucleus. The $^{14}$C population is a double population; some cells are labeled with $^{14}$C only and some with $^{14}$C + $^3$H, but the latter group cannot be measured with precision. The double labeling method has been used for the accurate determination of $t_s$ in mammalian and plant cell systems (Pilgrim and Maurer, 1963, 1965; Wimber and Quastler, 1963). Lala, Maloney and Patt (1965) measured $t_s$ for myeloid–erythroid precursors in canine marrow by (1) an *in vivo–in vitro* procedure, by pulse-labeling with $^3$HTdR *in vivo* followed at a short interval by relabeling *in vitro* with $^{14}$CTdR, and (2) an *in vitro–in vitro* procedure, by labeling with $^3$HTdR *in vitro* followed by relabeling with $^{14}$CTdR *in vitro*. Lala (1968) has used the *in vivo–in vitro* double-labeling procedure to obtain data for the measurement of the S period in growing populations of Ehrlich ascites tumor cells in mice.

### RESULTS

Fig. 1 is an autoradiograph of cells from a carcinoma of the bronchus in a 62 year old man. The $^{14}$C-labeled cell has a spray of grains in a number of planes around the nucleus. The cell labeled with $^3$H only has grains in one plane directly over the nucleus. Table I lists the values in the different human cell populations

TABLE I.—*Analysis of Human Cell Population Kinetics Using $^3$HTdR, $^{14}$CTdR Double Labeling Method*

| | Cell population | N | $N_s$ | LI | $N_{14C}$ | $N_{3H}$ | $t_s$ (hours) | T (hours) |
|---|---|---|---|---|---|---|---|---|
| 1. | Trachea normal | 2413 | 70 | 2·9 | 65 | 5 | 13·0 | 448·3 |
| 2. | Carina normal | 2139 | 39 | 1·8 | 36 | 3 | 12·0 | 666·7 |
| 3. | Bronchus normal | 2391 | 122 | 5·1 | 112 | 10 | 11·2 | 219·6 |
| 4. | Bronchus normal | 2172 | 100 | 4·6 | 92 | 8 | 11·5 | 250·0 |
| 5. | Larynx squamous cell papilloma | 2722 | 283 | 10·4 | 266 | 17 | 15·6 | 120·0 |
| 6. | Larynx squamous cell papilloma | 2839 | 287 | 10·1 | 271 | 16 | 16·9 | 133·1 |
| 7. | Larynx squamous cell papilloma | 2920 | 164 | 5·6 | 151 | 13 | 11·6 | 168·2 |
| 8. | Larynx carcinoma | 2698 | 208 | 7·7 | 199 | 9 | 22·1 | 204·5 |
| 9. | Larynx carcinoma | 2297 | 158 | 6·8 | 150 | 8 | 18·8 | 214·8 |
| 10. | Bronchus carcinoma | 2430 | 153 | 6·3 | 146 | 7 | 20·9 | 260·6 |
| 11. | Bronchus carcinoma | 2177 | 194 | 8·9 | 186 | 8 | 23·3 | 196·4 |
| 12. | Esophagus carcinoma | 2674 | 211 | 7·9 | 203 | 8 | 25·4 | 250·7 |
| 13. | Esophagus carcinoma | 2194 | 161 | 7·3 | 154 | 7 | 22·0 | 235·0 |

for $t_s$ and T, determined by analysis of cell population kinetics using the double label method. The following observations may be noted. (1) The range of the duration of the S period is from ~11–25 hours. (2) The $t_s$ values for normal tissues of the upper respiratory epithelium is from ~11–13 hours. (3) The $t_s$ values for malignant tumor cells are greater (range, 18·8–25·4 hours) than for benign tumor cells (range, 11·6–16·9 hours). (4) The potential T values for the malignant tumor cells are greater (range, 214·8–260·6 hours) than for benign tumor cells (range, 120·0–168·2 hours).

### DISCUSSION

If it is assumed that there is little variation in the flow rate of cells entering into and leaving DNA synthesis, *i.e.* a constant flux of cells into and out of S, and that the *in vivo–in vitro* transformation of the tissue environment has not introduced

---

EXPLANATION OF PLATE

FIG. 1.—Autoradiograph of tissue from human carcinoma of bronchus using $^3$HTdR, $^{14}$CTdR double labeling sequence with 1 hour between labels. The $^{14}$C-labeled cell has a spray of grains in a number of planes around the nucleus. The cell labeled with $^3$H only has grains in one plane directly over the nucleus. NTB2 nuclear emulsion, exposure time 3 weeks, hematoxylin and eosin, ×2000.

1

Fabrikant.

perturbations within the system, then the duration of DNA synthesis can be measured. During the interval between labels, $^{3}$H-labeled cells pass out of DNA synthesis and their number will be proportional to the time between labels. All $^{14}$C-labeled cells were in the S phase and their number will be proportional to the duration of DNA synthesis. The duration of synthesis can be determined from $t_s/t_a = N_{14C}/N_{3H}$ in an asynchronously proliferating cell population with a relatively uniform age distribution of cells in the proliferative compartment. In addition, some information is available on the duration of the potential tissue doubling time from determination of the LI, where $LI = N_S/N$, and $T = \lambda(t_a + t_s)/LI$ or $T = \lambda(t_a + t_s)N/N_s$. $\lambda$ is a growth constant of proportionality and depends on the duration and position of DNA syntheis in the cell cycle and thus on the age distribution of the proliferating cells. The value of $\lambda$ must be determined from the appropriate phase distribution diagram for each proliferating population (Bresciani, 1968; Fabrikant and Wisseman, 1968; Johnson, 1961; Steel and Bensted, 1965). For linear growth, $\lambda = 1{\cdot}0$; this occurs in steady-state cell renewal systems. For exponential growth, $\lambda = \ln 2/t_c$; this may occur in human cell systems such as lymphomas and early metastatic growth in lymph nodes. If the rate of cell loss is $\delta N$, then the net rate of growth is $dN/dt = \lambda N - \delta N$. For the cell renewal system in the steady state, $dN/dt = 0$ and $\delta = \ln 2/t_c$. For tumor cell populations, $\lambda$ is $\sim 0{\cdot}75$–$0{\cdot}8$, based on very limited information available, and this value cannot be estimated with any precision (Fabrikant and Wisseman, 1968; Steel and Bensted, 1965; Steel, 1968). The analysis of human cell population kinetics determined from the double labeled autoradiographs (Table I) is based on $t_a = 1$ hour and $\lambda = 1{\cdot}0$ for linear growth and $\lambda = 0{\cdot}75$ for tumor growth. However, at present, the values assumed for $\lambda$ are poorly supported by sparse experimental evidence, and it is expected that estimates of T based in thymidine labeling indices and growth rate constants will require constant revision as new data become available.

In the past, the estimates of kinetic parameters in human tissues have been limited primarily to $^{3}$HTdR labeling indices following a single injection just before surgery; T values were usually calculated from labeling indices, assuming an arbitrary value for $t_s$ and all cells in the population proliferating, *i.e.* the growth fraction (Mendelsohn, 1962) is unity. Since the growth fraction varies in different tissues, and particularly among neoplasms, such estimates have been inaccurate. Recently, several normal patients and patients with leukemia, neoplastic effusions and solid tumors have been studied using serial sample techniques after $^{3}$HTdR-labeling *in vivo* (Bennington, 1969; Clarkson *et al.*, 1965; Lala *et al.*, 1965; Lipkin, Bell and Sherlock, 1963; Lipkin, Sherlock and Bell, 1963; Mauer and Fisher, 1963, 1966; Stryckmans *et al.*, 1966). The human data indicate $t_s$ values in some normal tissues and leukemic blast cells of $\sim$10–15 hours, and in neoplastic tissues, $\sim$20–30 hours. Until further data are available, $\sim$10–15 hours is the best approximation for average DNA synthesis time in the bone marrow precursor cells in man. These values compare favorably with those determined with the *in vitro* double labeling method reported here (Table I). In addition, $t_c$ and T values are much longer in man than in experimental animals.

The $^{14}$C-, $^{3}$HTdR double labeling technique can be used for determining $t_s$ by one double labeling sequence if it is assumed that (1) there is a constant flux of cells into and out of S, and (2) there is an asynchronous distribution of cells within the cell cycle. Normally, the procedure measures cell fluxes through DNA

synthesis in steady state renewal tissues, but corrections can be made for growing cell populations with characteristics between steady state and exponential growth. This can be done if there is random cell loss during the cycle or at the end of mitosis, and the growth fraction is known. Lala (1968) applied the analysis to the Ehrlich ascites tumor in mice, where the growth fraction declines with increasing tumor age due to transition from a dividing to a nondividing state occurring mostly at the end of mitosis (Lala *et al.*, 1965).

The double labeling method can be used to determine $t_c$ with precision only in the steady state renewal system which is dividing asynchronously, in which there is no cell loss during S, a growth fraction of unity, and a relatively narrow distribution of cell cycle times. However, the data on the LI and $t_s$ can predict a *potential* tissue doubling time, that is, the time for the cell population to double if there is no cell loss. Steel (1968) has demonstrated in 6 different experimental tumor cell systems in mammals that when the tumors were small, the measured volume doubling times *in vivo* were very similar to the potential doubling times determined from $^3$HTdR, LI and accurate values of $t_s$. As the tumors grew, however, changes in the sizes of the growth fraction and the continuous loss of cells through cell death, metastasis or emigration, gave rise to a difference between the median cell cycle time, the potential tumor doubling time, and the actual doubling time. The effect of these processes—changes in the growth fraction and cell loss—on the rate of growth in human tumors is not as yet known.

This work was supported by a Research Grant from the James Picker Foundation on recommendation of the Committee on Radiology NAS–NRC. I am indebted to Dr. Russell H. Morgan, Professor of Radiological Science at The Johns Hopkins University, for his constant support and encouragement. I wish to thank Miss Mary J. Vitak and Dr. Bernard Marsh for their expert assistance.

### REFERENCES

Baserga, R.—(1965) *Cancer Res.*, **25**, 581.—(1968) *Cell and Tissue Kinetics*, **1**, 167.
Bennington, J. L.—(1969) *Cancer Res.*, **29**, 1082.
Bresciani, F.—(1968) *Eur. J. Cancer*, **4**, 343.
Clarkson, B., Ota, K., Ohita, T. and O'Connor, A.—(1965) *Cancer, N.Y.*, **18**, 1189.
Fabrikant, J. I. and Cherry, J.—(1969) *Ann. Otol. Rhinol. Lar.*, **78**, 326.
Fabrikant, J. I. and Wisseman, III, C. L.—(1968) *Radiology*, **90**, 361.
Fabrikant, J. I., Wisseman, III, C. L. and Vitak, M. J.—(1969) *Radiology*, **92**, 1309.
Howard, A. and Pelc, S. R.—(1953) *Hereditas*, Suppl. 6, p. 261.
Johnson, H. A.—(1961) *Cytologia*, **26**, 32.
Lajtha, L. C., Oliver, R. and Ellis, F.—(1954) *Br. J. Cancer*, **8**, 367.
Lala, P. K.—(1968) *Expl Cell Res.*, **50**, 461.
Lala, P. K., Maloney, M. A. and Patt, H. M.—(1965) *Expl Cell Res.*, **38**, 626.
Lipkin, M., Bell, B. and Sherlock, P.—(1963) *J. clin. Invest.*, **42**, 767.
Lipkin, M., Sherlock, P. and Bell, B.—(1963) *Gastroenterology*, **45**, 721.
Mauer, A. M. and Fisher, V.—(1963) *Nature, Lond.*, **197**, 574.—(1966) *Blood*, **28**, 428.
Mendelsohn, M. L.—(1962) *J. natn. Cancer Inst.*, **28**, 1015.
Pilgrim, C. and Maurer, W.—(1963) *Naturwissenschaften*, **199**, 863.—(1965) *Expl Cell Res.*, **37**, 183.
Prescott, D. M.—(1968) *Cancer Res.*, **28**, 1815.
Quastler, H.—(1963) ' Cell proliferation '. Oxford (Blackwell), p. 18.
Steel, G. G.—(1968) *Cell and Tissue Kinetics*, **1**, 193.

STEEL, G. G. AND BENSTED, J. P. M.—(1965) *Eur. J. Cancer*, **1**, 275.
STRYCKMANS, P., CRONKITE, E. P., FACHE, J., FLIEDNER, T. M. AND RAMOS, J.—(1966), *Nature, Lond.*, **211**, 717.
WIMBER, D. E. AND QUASTLER, H.—(1963) *Expl Cell Res.*, **30**, 8.